# Collagen IV-β1-Integrin Influences INS-1 Cell Insulin Secretion *via* Enhanced SNARE Protein Expression

**DOI:** 10.3389/fcell.2022.894422

**Published:** 2022-04-28

**Authors:** Malina Barillaro, Meg Schuurman, Rennian Wang

**Affiliations:** ^1^ Children’s Health Research Institute, London, ON, Canada; ^2^ Department of Physiology and Pharmacology, University of Western Ontario, London, ON, Canada

**Keywords:** B1 integrin, collagen IV, INS-1 cells, exocytotic proteins, glucose-stimulate insulin secretion

## Abstract

β1-integrin is a key receptor that regulates cell-ECM interactions and is important in maintaining mature beta-cell functions, including insulin secretion. However, there is little reported about the relationship between ECM-β1-integrin interactions and exocytotic proteins involved in glucose-stimulated insulin secretion (GSIS). This study examined the effect of collagen IV-β1-integrin on exocytotic proteins (Munc18-1, Snap25, and Vamp2) involved in insulin secretion using rat insulinoma (INS-1) cell line. Cells cultured on collagen IV (COL IV) had promoted INS-1 cell focal adhesions and GSIS. These cells also displayed changes in levels and localization of β1-integrin associated downstream signals and exocytotic proteins involved in insulin secretion. Antibody blocking of β1-integrin on INS-1 cells cultured on COL IV showed significantly reduced cell adhesion, spreading and insulin secretion along with reduced exocytotic protein levels. Blocking of β1-integrin additionally influenced the cellular localization of exocytotic proteins during the time of GSIS. These results indicate that specific collagen IV-β1-integrin interactions are critical for proper beta-cell insulin secretion.

## Introduction

Investigation of extracellular matrix (ECM) components for beta-cell function is crucial to optimizing cell-based therapies for the treatment of diabetes. ECM-cell interactions are critical for proper cell function, and integrins are a common component in ECM-cell interactions (Hynes, 2002). β1-integrin is the most prevalent β subunit pairing with numerous α subunits. This enables numerous ligand pairings and cellular processes (Bosco et al., 2000; Plow et al., 2000; Stupack and Cheresh, 2002; Wang et al., 2005; Howe and Addison, 2012; Arous and Wehrle -Haller, 2017). In beta-cells, β1-integrin is well described in cell functions including cell adhesion, survival, development, and insulin secretion (Wang et al., 2005; Yashpal et al., 2005; Wang and Wang, 2009; Diaferia et al., 2013; Peart et al., 2017). This is achieved *via* multiple signaling pathways mainly stemming from focal adhesion sites, where focal adhesion kinase (FAK) is a common primary signaling molecule. The majority of research identifies FAK to regulate insulin secretion via its regulation of the actin cytoskeleton (Cai et al., 2012). However, alternative roles for FAK in insulin secretion have been identified. BCL-2-associated athanogene 3 (Bag3), a downstream effector of FAK, impacts insulin secretion. Iorio et al. demonstrated that, in an unstimulated state, Bag3 is bound to Snap25, preventing it from interacting with v-SNAREs (vesicle-Soluble N-ethylmaleimide Sensitive Factor Attachment Protein Receptors) such as Vamp2, and in a glucose-stimulated state FAK phosphorylates Bag3 releasing it from Snap25 facilitating SNARE complex formation and insulin secretion (Iorio et al., 2015).

There are various ECMs present in and around islets for β1-integrin activation (Wang et al., 2005; Arous and Wehrle-Haller, 2017). Our previous studies have demonstrated a crucial role for collagen IV-β1-integrin interactions in the promotion of insulin secretion, among other enhancements, from rat insulinoma line, INS-1 cells (Krishnamurthy et al., 2008; Krishnamurthy et al., 2011). Although the pathway of β1-integrin activation to enhanced insulin secretion is well studied, the influence of β1-integrin on the exocytotic machinery, specifically SNARE proteins, involved has not been fully explored. The impact of integrin activation on exocytotic proteins has been demonstrated in neurons that have similar exocytotic mechanisms to beta-cells. For example, laminin-mediated β1-integrin activation was shown to be responsible for cytoskeletal rearrangement and controlling VAMP7-regulated exocytosis and neurite development of cortical neurons in mice (Gupton and Gertler, 2010). Following inhibition of upstream signaling molecules involved in the β1 integrin-signaling pathway, neuritogenesis was rescued by overexpressing VAMP7 (Gupton and Gertler, 2010). The current study aimed to explore the relationship between collagen IV-β1-integrin interaction and exocytotic proteins expression and localization in beta cell insulin secretion. Here, we showed the influence of collagen IV and β1-integrin interactions on INS-1 cell exocytotic proteins and the subsequent augmentation in glucose-stimulated insulin secretion (GSIS). INS-1 cells cultured on COL IV displayed increased insulin secretion that depended on increased cell focal adhesion, activation of β1-integrin and downstream FAK/Bag3 phosphorylation, and elevated SNARE proteins and their cellular colocalization. Blockade of β1-integrin diminished INS-1 cell interaction with COL IV and subsequent changes in SNARE protein expression and colocalization that enhanced insulin secretion, indicating that collagen IV-β1-integrin interactions are critical for proper beta-cell insulin secretion.

## Materials and Methods

### Cell Culture

INS-1 832/13 cells (passages 4–30; a gift from Dr. Christopher Newgard, Duke University Medical Center, United States) were cultured in RPMI-1640 media with L-glutamine (Gibco, Amarillo, Texas, United States) containing 10% fetal bovine serum (FBS), 1 mmol/L sodium pyruvate (Invitrogen, Burlington, ON, Canada), 10 mmol/L HEPES and 50 μmol/L β-mercaptoethanol (Sigma, St. Louis, MO, United States) (Krishnamurthy et al., 2008). At 90% confluency, INS-1 were used for experimental studies. For collagen IV matrix study (COL IV), 12- or 96-well tissue culture plates, or cell culture chamber slides (Fisher Scientific, Ottawa, ON, Canada) were precoated with either collagen IV matrix protein at 5 µg per cm^2^ (working solution as 20 μg/ml in 0.05M HCl; Santa Cruz Biotechnology Inc., Dallas, TX, United States) or 1% bovine serum albumin (BSA; Sigma) as control (CTRL). INS-1 cell were placed on precoated plates and cultured in serum-free RPMI medium plus 1% BSA for 24 h. Three to six cell passages were used for each set of experiments, representing *n* = 3–6. For functional blockade of β1-integrin, INS-1 cells were pretreated with hamster anti-rat β1-integrin antibody (CD29, 5 μg/ml) (anti-β1), hamster IgM isotype-matched negative control (5 μg/ml) (IgM) (BD Biosciences, Mississauga, ON, Canada) or untreated in serum free media as control group (Ctrl) for 1 h prior to being plated on collagen IV pre-coated plates or chamber slides and cultured for 24 h. Cells were harvested and processed for protein extraction or fixed for immunocytochemistry studies.

### Adhesion and Spreading Assay

Cells were plated on 96-well tissue culture plates pre-coated with collagen IV matrix protein or BSA. To analyze adhesion, cells were cultured in serum-free media for 3 h, wells were rinsed twice using 1x PBS to remove non-adhered cells, and six random fields were imaged per well using a Leica DMIRE2 microscope (Leica Microsystems) at 40x magnification. After 24 h, cells were again imaged to analyze cell spreading. Cells that adhered or spread were counted and normalized to control. Data is expressed as fold change versus control. Each experiment was performed in triplicate with six biological repeats per group.

### Glucose-Stimulated Insulin Secretion Assays

INS-1 cells (1x10^5^) were seeded and cultured on collagen IV or control for 24 h followed by media collection to determine basal insulin secretion. Wells were gently rinsed twice with no glucose RPMI-1640 (Sigma) plus 0.5% BSA followed by incubation in RPMI-1640 plus 0.5% BSA with 2.2 mmol/L glucose then 22 mmol/L glucose for 1 hour each. Media was collected after each treatment to analyze insulin secretion in response to glucose stimulation. Cells were then harvested to determine insulin content.

For time dependent glucose-stimulated insulin secretion (GSIS), INS-1 cells (1.5x10^5^) were plated on cell culture chamber slides pre-coated with collagen IV or BSA for 24 h. Basal media was collected. INS-1 cells were rinsed twice with no glucose RPMI-1640 plus 0.5% BSA, followed by one of four glucose stimulation conditions: followed by one of four glucose stimulation conditions: (1) 2.2 mmol/L glucose for 30 minutes (L30), (2) 22 mmol/L glucose for 5 minutes (H5), (3) 30 minutes (H30), or (4) 60 minutes (H60). Media were collected at each stimulation condition and cells were immediately fixed in 4% PFA followed by immunofluorescent staining for SNARE protein localization analysis.

Insulin concentrations were determined by a Stellux chemiluminescent high range rodent insulin ELISA kit (Alpco, Salem, NH, United States). Static GSIS stimulation index was calculated for each group to account for differences in adhesion and expressed as the ratio of insulin secretion at 22 mM over 2.2 mM glucose stimulation (Krishnamurthy et al., 2008). Insulin content was normalized to total cell protein concentration. Data are expressed as ng/ml or as ng/mg protein (Krishnamurthy et al., 2008). For time dependent GSIS, data are expressed as rate of insulin secretion at ng/ml per minute. Each experiment was performed in technical triplicates with 3–5 biological repeats per group.

### Immunofluorescent Analysis

Cultured cells were fixed in 4% PFA, embedded in 2% agarose gel and process into tissue blocks (Krishnamurthy et al., 2008). 4 µm sections were taken and stained with appropriate dilutions of primary antibodies as listed in a Supplementary Table S1 followed by incubation with secondary antibodies conjugated with either fluorescein isothiocyanate (FITC) or tetramethyl rhodamine isothiocyanate (TRITC) (Jackson Immunoresearch, West Grove, PA). Nuclei were counterstained with DAPI (Sigma). Positive staining images were captured using a Nikon Eclipse Ti2 confocal microscope (Nikon, Mississauga, ON) set to 60x magnification with oil. Imaging was captured at 6–7 random areas per section with a minimum of three biological repeats per group. Displayed images are representative of staining found in each experimental group.

To assess alterations in the structural organization of focal adhesion contacts, an Actin Cytoskeleton and Focal Adhesion Staining kit was used containing TRITC-conjugated Phalloidin and a monoclonal antibody for Vinculin (Krishnamurthy et al., 2008).

After time-dependent GSIS, INS-1 cells in chamber slides were fixed immediately, incubated in 0.2% Triton for 30 min, then stained and imaged as described above in order to analyze for changes in protein intensity and localization. Displayed images are representative of staining found in each group.

### Protein Extraction and Western Blotting

INS-1 cell protein was extracted by sonicating cells in lysis buffer containing Nonident-P40, phenylmethylsolfonyl fluoride, sodium orthovanadate (Sigma) and complete protease inhibitor cocktail tablet (Roche; Mississauga, ON, Canada). Equal amounts (25 µg) of lysate proteins from each experimental group was separated by either 10 or 12% sodium dodecyl sulfate-polyacrylamide gel electrophoresis (SDS-PAGE). Following separation, proteins were transferred to nitrocellulose membrane (Bio-Rad Laboratories Inc.), and membranes were washed in Tris buffer-saline with 0.1% Tween-20 (TBST; Sigma) followed by blocking in 5% nonfat dry milk. Immunoblotting was performed with appropriate dilutions of primary antibodies as listed in Supplementary Table S1 overnight at 4°C, followed by application of appropriate horse-radish peroxidase (HRP)-conjugated secondary antibodies. Proteins were detected by chemiluminescent detection (ECL, PerkinElmer, Waltham, MA, United States). Protein of interest were visualized using a Versadoc version 4.6.9 imaging system, and densitometric quantification of bands was determined by Image Lab software (Bio-Rad Laboratories Inc.). Reference protein (GAPDH) and total proteins were used to normalize protein bands of interest, and data is expressed as fold-change from control (Krishnamurthy et al., 2008; Riopel et al., 2011).

### Statistical Analysis

Data are expressed as means ± SEM. Statistical significance was determined using a paired t-test or repeated measures one-way ANOVA followed by Tukey’s post-hoc test, if significant. Differences were considered statistically significant when *p* < 0.05.

## Results

### Collagen IV Enhances INS-1 Cell Adhesion, Spreading, Focal Adhesion and Insulin Secretion

**FIGURE 1:**
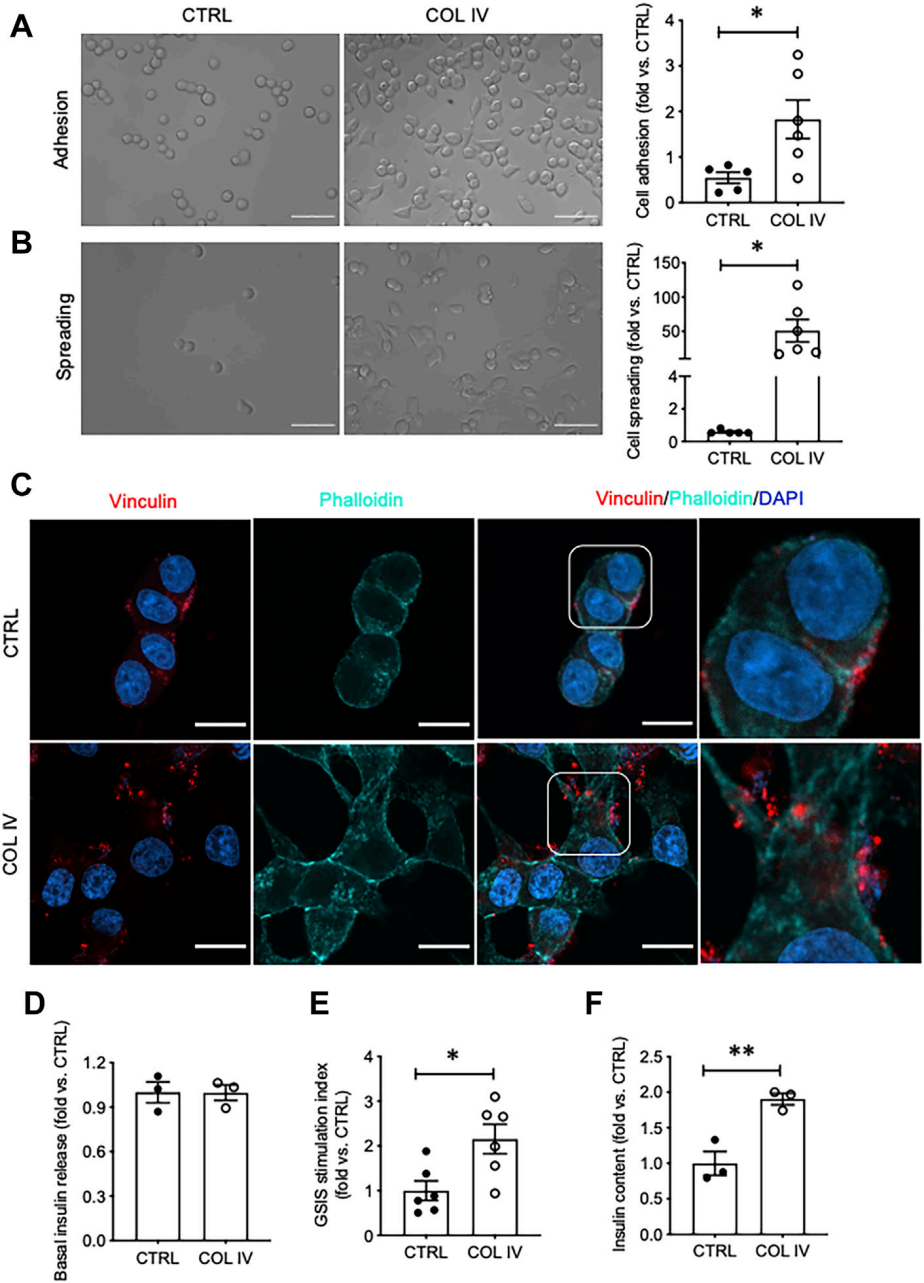
Collagen IV enhances INS-1 cell adhesion, spreading, focal adhesion, and insulin secretion. Phase contrast images of INS-1 cells cultured on collagen IV (COL IV) or BSA-coated control (CTRL) and quantification of cell **(A)** adhesion and **(B)** spreading. Scale bar: 50 µm. *p < 0.05 vs. CTRL, data are expressed as fold change vs. control (mean ± SEM, *n* = 6 experiments/group). **(C)** Immunostaining for vinculin (red) and F-actin (cyan) of INS-1 cells cultured on COL IV- and CTRL-coated chamber slides for 24 h. Nuclei are counterstained with DAPI, and magnified images are highlighted. Scale bar: 10 µm. **(D)** Basal insulin release, **(E)** glucose stimulated insulin secretion (GSIS) stimulation index and **(F)** cellular insulin content from INS-1 cells cultured on COL IV or CTRL. *p < 0.05, **p < 0.01 vs. CTRL. Data are expressed as mean ± SEM (*n* = 3–6 experiments/group).

The effect of collagen IV on INS-1 cell adhesion and spreading was first tested, as these are early visual indicators of ECM-cell interactions. Visually and quantitatively, collagen IV provided robust increases in both adhesion and spreading compared to control (*p* < 0.05, Figures 1A,B). Culturing INS-1 cells on collagen IV provided major differences in focal adhesions compared to control, determined by phalloidin and vinculin staining (Figure 1C). Control cells were round with weak actin and vinculin staining, while cells in the collagen IV group were stretched, and had intense actin at the periphery with increased clusters of vinculin indicating an increase in focal adhesion (Figure 1C). Although basal insulin secretion was not found to differ between groups (Figure 1D), both GSIS stimulation index and insulin content was found to be significantly increased in the collagen IV group versus control (*p* < 0.05, *p* < 0.01, Figures 1E,F).

### Collagen IV Impacts β1-Integrin Signaling Associated With Insulin Secretion

**FIGURE 2:**
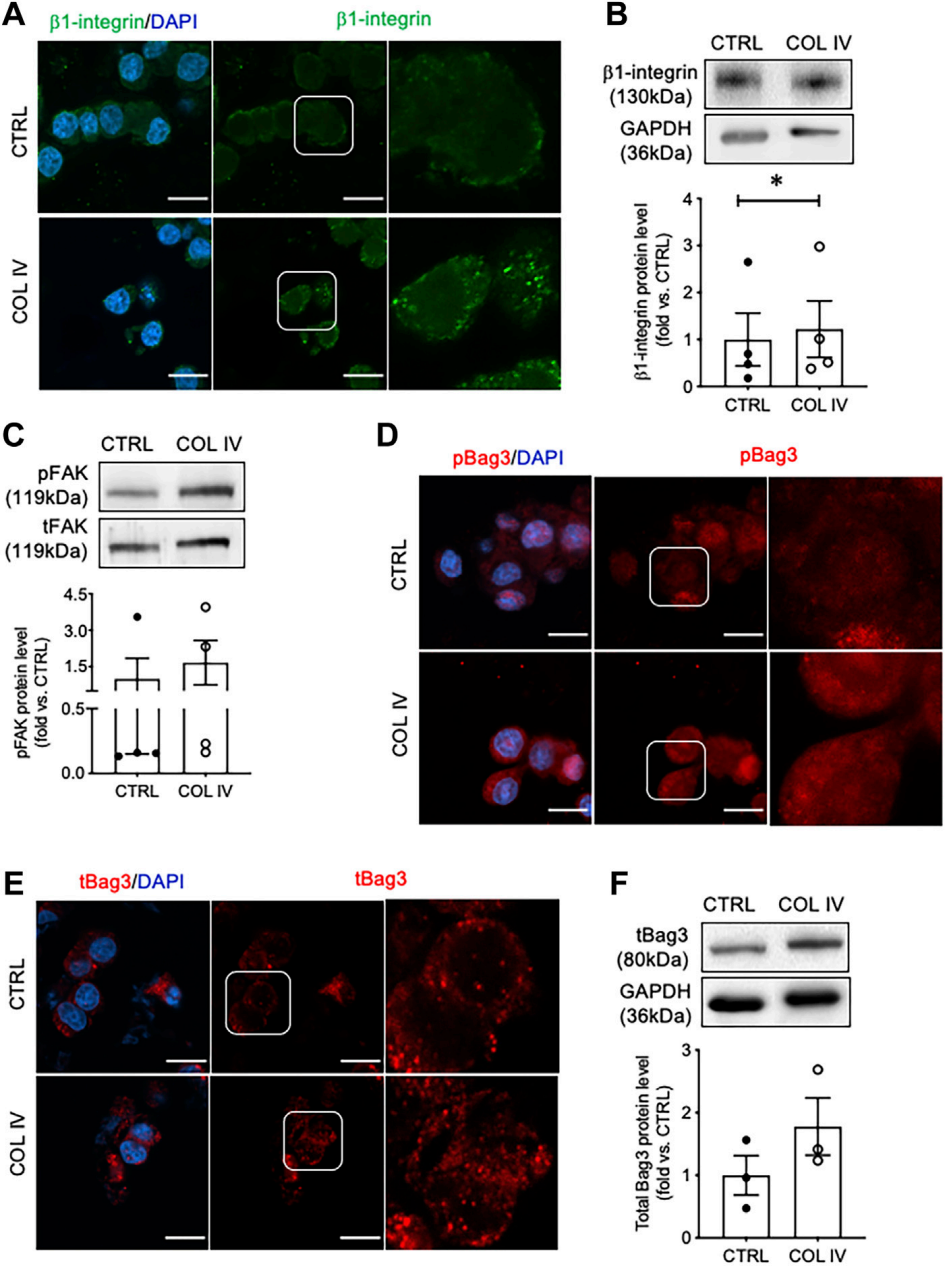
Collagen IV enhances β1-integrin, FAK and Bag3 phosphorylation in INS-1 cells. Representative **(A)** β1-integrin (green) immunostaining and **(B)** blotting image with data analysis; **(C)** phosphorylated FAK (pFAK) and total FAK (tFAK) blotting image and quantified data; representative immunostaining images for **(D)** phosphorylated Bag3 (pBAG3, red), and **(E)** total Bag3 (tBag3, red) and **(F)** tBag3 blotting image and blot data quantification in INS-1 cells cultured on collagen IV (COL IV) or BSA-coated control (CTRL). Nuclei are counterstained with DAPI, and magnified images are highlighted. Scale bar: 10 µm. Data are expressed as mean ± SEM (*n* = 3–4 experiments/group).

To assess signaling associated with insulin secretion initiated by collagen IV-INS-1 cell interactions, β1-integrin, FAK, and Bag3 were examined. A notable increase in intensity and membrane-associated puncta of β1-integrin was observed in the collagen IV group compared to control (Figure 2A). While no significant changes in total protein levels were found (Figure 2B). FAK is a known downstream signaling molecule from β1-integrin and was found to have elevated phosphorylation in the COL IV group compared to control, although not statistically significant (Figure 2C). Bag3, a protein phosphorylated by FAK and shown to be involved in regulation of insulin exocytosis (Iorio et al., 2015), had a high density of phosphorylated staining (Figure 2D), a minor increase in puncta of total Bag3 staining (Figure 2E), and a 1.4-fold increase in total Bag3 protein levels (Figure 2F) in COL IV cells when compared to control, but statistical significance was not reached due to a large variation between sets of experimental repeats.

### Collagen IV Influences Expression of SNARE Proteins Required for Insulin Secretion

**FIGURE 3:**
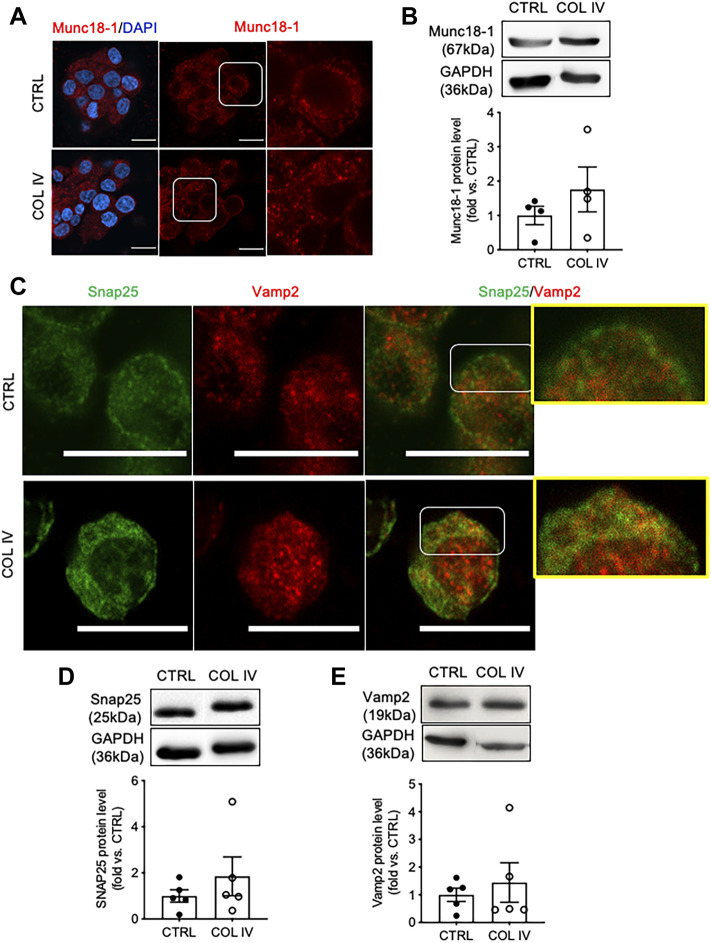
Collagen IV impacts SNARE protein level and colocalization in INS-1 cells. Representative **(A)** Munc18-1 (red) immunostaining and **(B)** blotting images and quantified data. Double immunofluorescence staining for **(C)** Snap25 (green) and Vamp2 (red); representative blotting images and data analysis for **(D)** Snap25 and **(E)** Vamp2 in INS-1 cells cultured on collagen IV (COL IV) or BSA-coated control (CTRL) for 24 h. Nuclei are counterstained with DAPI, and magnified images are highlighted. Scale bar: 10 µm. Data are expressed as mean ± SEM (*n* = 4–5 experiments/group).

To examine whether collagen IV induced increased in insulin secretion is linked to the alteration of SNARE proteins, Munc18-1, Snap25, and Vamp2 were examined. INS-1 cells cultured on collagen IV displayed increased cytoplasmic puncta of Munc18-1 (Figure 3A) with 1.8-fold increase in protein level compared to control (Figure 3B). Cells in the COL IV group also showed increased intensity of Snap25 in the cytoplasm and membrane, relative high staining signals of Vamp2 in the cytoplasm, and an increase in Snap25 and Vamp2 colocalization, as showed in yellow, compared to control (Figure 3C). Both Snap25 and Vamp2 protein levels were elevated in COL IV cells at 1.9- or 1.5-fold when compared to control cells (Figures 3D,E). However, a large variation between sets of experiment repeats made the western blot analysis data not reach statistical significance.

### Collagen IV Enhances Colocalization of Snap25 and Vamp2, and Insulin Release Rate During GSIS

**FIGURE 4:**
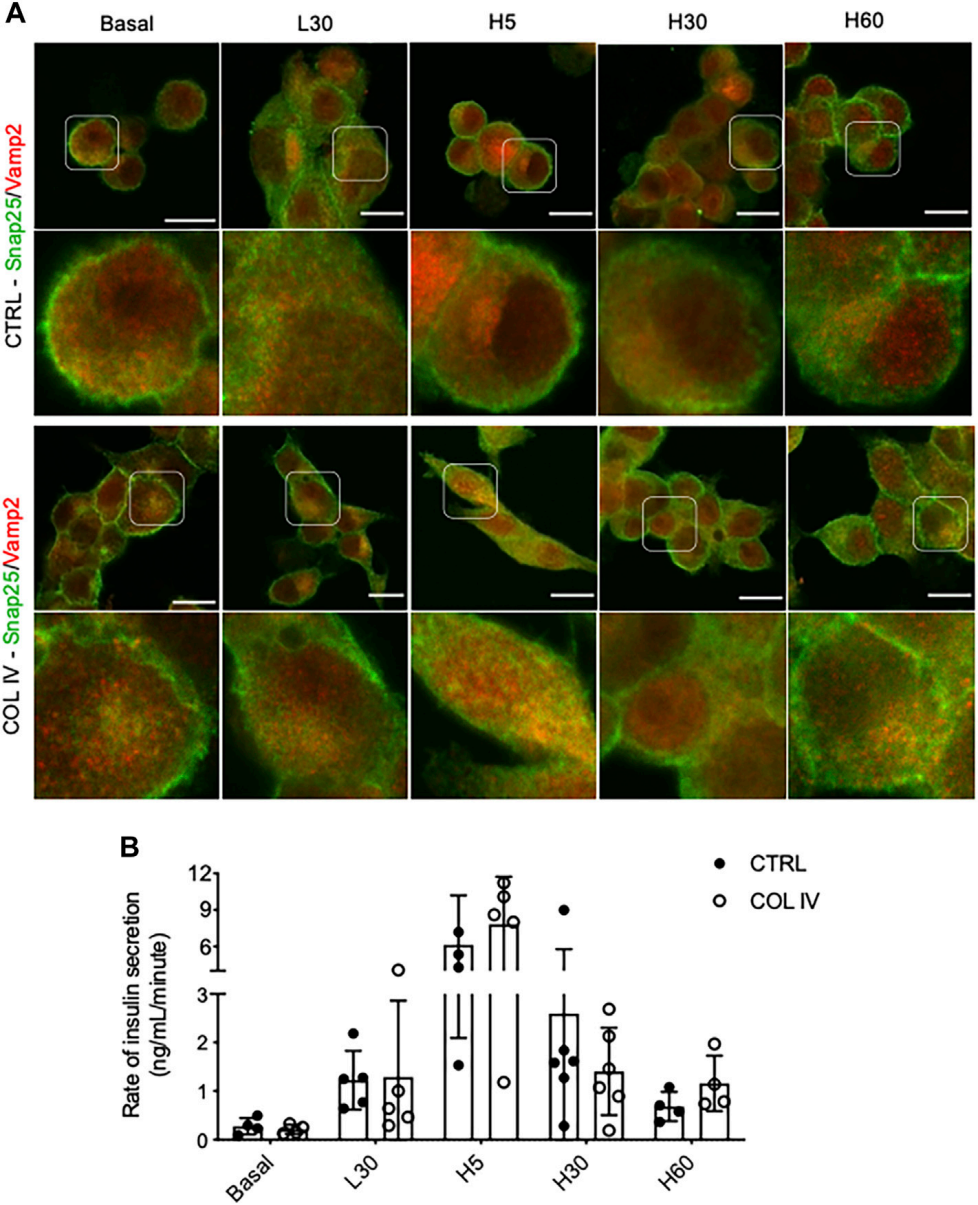
Collagen IV enhances Snap25 and Vamp2 colocalization and rate of insulin secretion during GSIS in INS-1 cells. Representative double immunostaining of **(A)** Snap25 (green) and Vamp2 (red) in INS-1 cells cultured on collagen IV (COL IV) or BSA-coated control (CTRL) in basal, low glucose (2.2 mmol) for 30 min (L30), high glucose (22 mmol) for 5 min (H5), 30 min (H30), and 60 min (H60). Nuclei are counterstained with DAPI, and magnified images are highlighted. Scale bar: 10 µm. **(B)** Rate of insulin secretion in each of the treatment conditions. Data are expressed as ng per mL per minute (mean ± SEM; *n* = 4–6 experiments/group).

To examine time and glucose-dependent changes of Snap25 and Vamp2 colocalization, double immunofluorescence staining for Snap25 and Vamp2 were performed in INS-1 cells cultured on chamber slides following varied conditions of glucose stimulation. At basal and GSIS conditions, cells cultured on collagen IV showed Snap25 to be more defined membrane staining than control (Figure 4A). A similar colocalization of Snap25 and Vamp2 at basal and following 30 min of low glucose stimulation (L30) was observed in both COL IV and control groups, which was correlated with similar rates of insulin release (Figure 4B). An increase of Snap25 and Vamp2 colocalization at cellular membrane and cytoplasm was found in COL IV cells under 5 min of high glucose stimulation (H5; Figure 4A), with elevated rate of insulin secretion (COL IV 8 ng/mL/minute vs. CTRL 6 ng/mL/minute) (Figure 4B). There was no difference for Snap25 and Vamp2 expression and colocalization at 30 min of high glucose stimulation (H30; Figure 4A) with similar insulin secretion rates (COL IV 1.4 ng/mL/minute vs. CTRL 1.6 ng/mL/minute) between the experimental groups (Figure 4B). At 60 min of high glucose stimulation (H60), cells cultured on COL IV had a higher rate of insulin secretion (1.2 ng/mL/minute) compared to control (0.7 ng/mL/minute) (Figure 4B), along with slightly enhanced Snap25 and Vamp2 expression and colocalization (Figure 4A).

### Blocking β1-Integrin Diminishes Collagen IV Induced INS-1 Cell SNARE Protein Expression and Insulin Secretion

**FIGURE 5:**
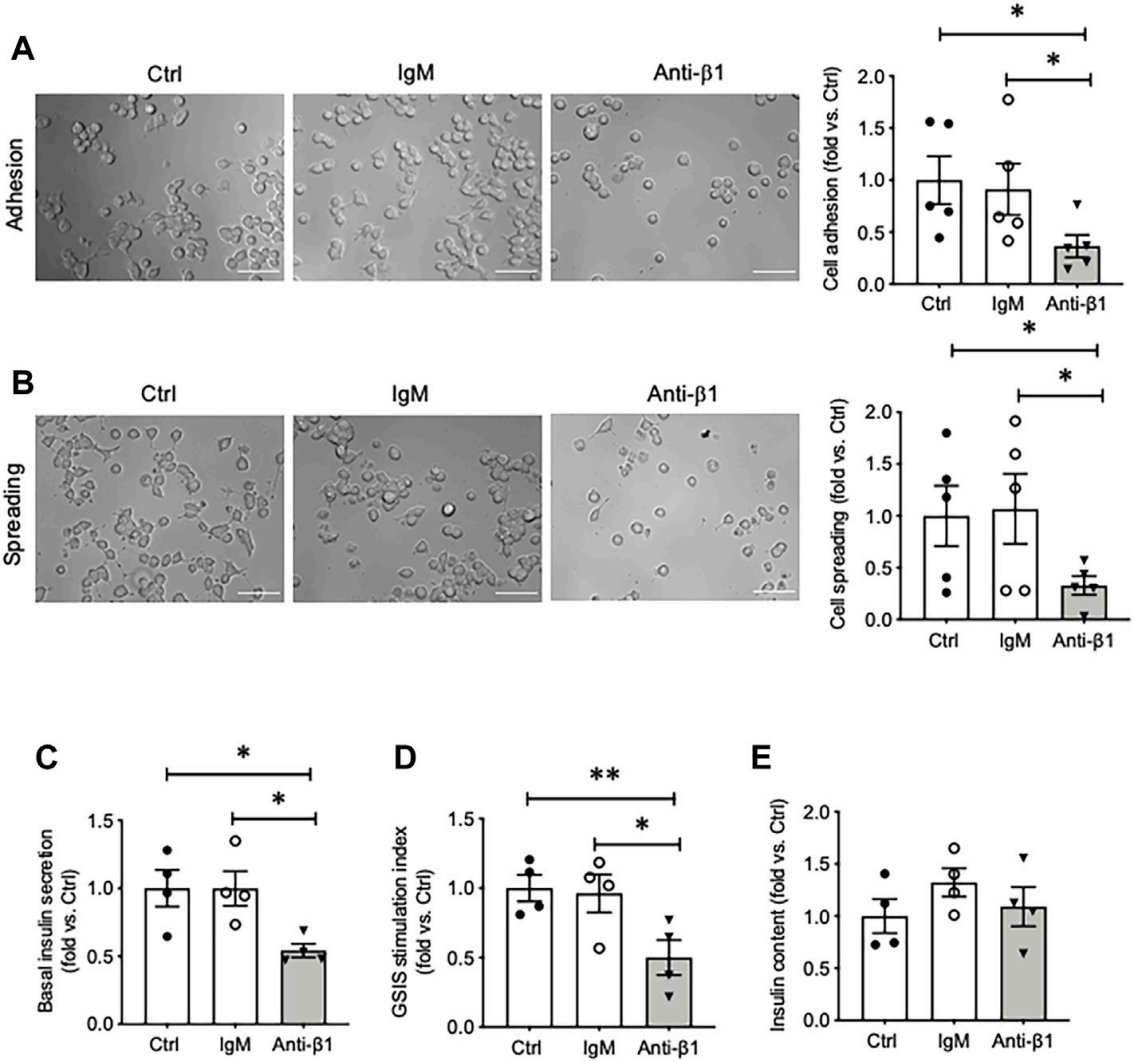
Blocking β1-integrin reduces INS-1 cell adhesion, spreading, basal and glucose stimulated insulin secretion. Phase contrast images and quantification analysis of cell **(A)** adhesion and **(B)** spreading. Scale bar: 10 µm. Data are expressed as fold change vs. untreated control (mean ± SEM; *n* = 5 experiments/group). **(C)** Basal insulin release, **(D)** glucose stimulated insulin secretion (GSIS) stimulation index, and **(E)** cellular insulin content from INS-1 cells cultured on collagen IV for 24 h treated with anti-β1 or IgM, or untreated control. *p < 0.05, **p < 0.01 vs. Ctrl. Data are expressed as mean ± SEM (*n* = 4 experiments/group).

To determine if the enhancement of SNARE protein and insulin secretion in cells cultured on collagen IV was due to β1-integrin signaling, INS-1 cells were pretreated with a β1-integrin blocking antibody. When compared to untreated and IgM-matched controls, INS-1 cells under functional blockade of β1-integrin treatment showed decreased cell adhesion and spreading on collagen IV-coated plates (Figures 5A,B). Blocking β1-integrin also caused significant reduction of basal insulin secretion (∼45%) (*p* < 0.05, Figure 5C) and GSIS stimulation index (∼50%) (*p* < 0.01 vs. Ctrl, *p* < 0.05 vs. IgM, Figure 5D), but no change in cellular insulin content compared to controls (Figure 5E).

**FIGURE 6:**
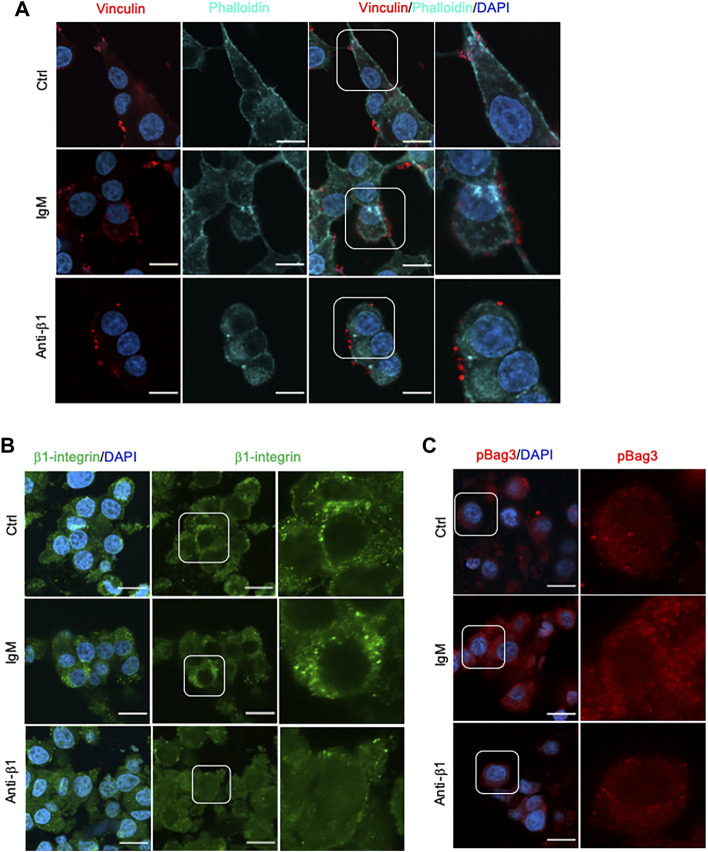
Blocking β1-integrin diminished focal adhesion, β1-integrin and BAG3 phosphorylation immunofluorescence signals. Representative immunostaining images of **(A)** vinculin (red) and F-actin (cyan), **(B)** β1-integrin (green), and **(C)** pBAG3 (red) in INS-1 cells treated with anti-β1, IgM, or untreated control (Ctrl) cultured on collagen IV for 24 hrs. Nuclei are counterstained with DAPI, and magnified images are highlighted. Scale bar: 10 µm.

**FIGURE 7:**
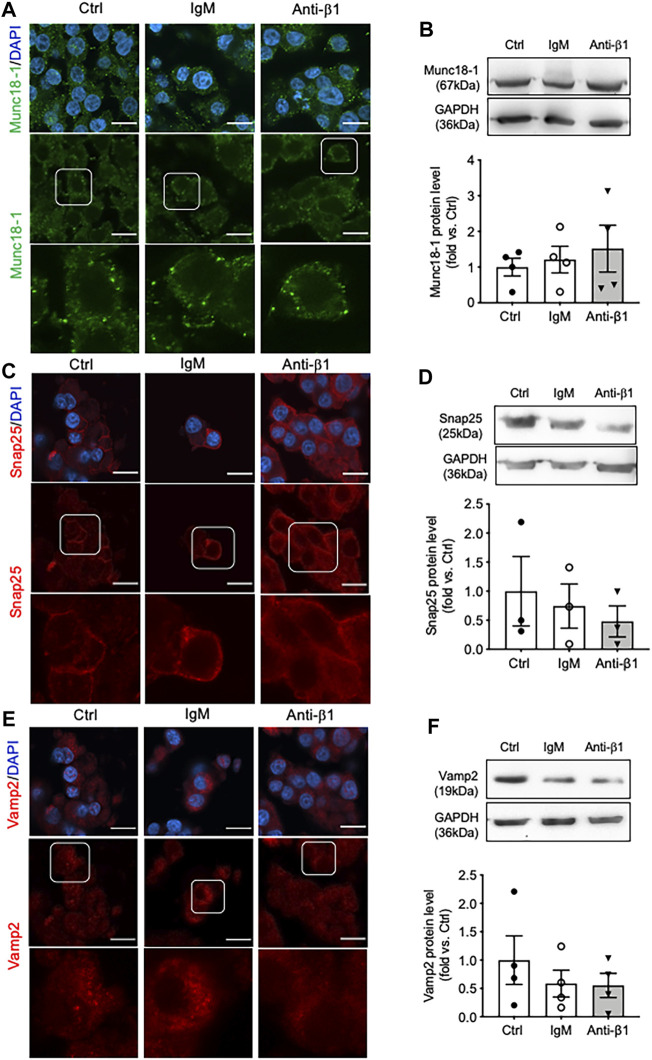
Blocking β1-integrin reduces Snap25, Vamp2, but not Munc18-1, protein level and immunostaining. Representative immunostaining images of **(A)** Munc18-1 (green), **(C)** Snap25 (red) and **(E)** Vamp2 (red) in INS-1 cells treated with anti-β1, IgM, or untreated control (Ctrl) cultured on collagen IV for 24 h. Nuclei are counterstained with DAPI, and magnified images are highlighted. Scale bar: 10 µm. Representative western blot images and data analysis for **(B)** Munc18-1, **(D)** Snap25, and **(F)** Vamp2 protein levels. Data are expressed as mean ± SEM (*n* = 3–4 experiments/group).

**FIGURE 8:**
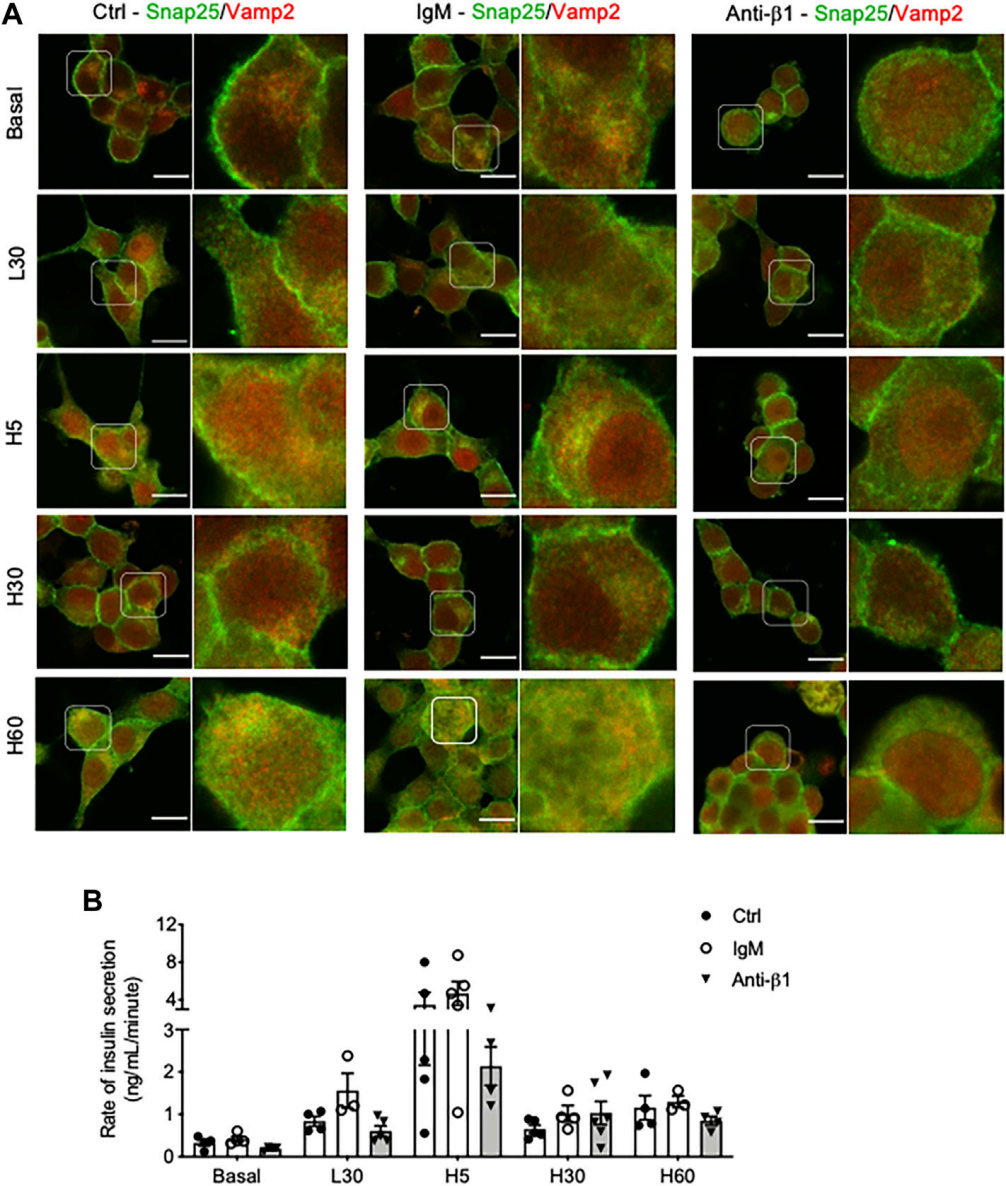
Blocking β1-integrin diminishes INS-1 cell Snap25/Vamp2 colocalization during GSIS and rate of insulin secretion throughout GSIS. Representative double immunostaining of **(A)** Snap25(green) and Vamp2 (red) in INS-1 cells cultured on collagen IV and treated with anti-β1, IgM, or untreated control (Ctrl) for 24hrs following one of five conditions: no treatment (basal) low glucose (2.2 mmol) for 30 min (L30), high glucose (22 mmol) for 5 min (H5), 30 min (H30), and 60 min (H60). Nuclei are counterstained with DAPI, and magnified images are highlighted. Scale bar: 10 µm. **(B)** Rate of insulin secretion (ng/mL/minute) in each of the treatment groups. Data are expressed as mean ± SEM (*n* = 3–5 experiments/group).

Examining INS-1 cell focal adhesions, control cells were highly spread and displayed intense actin filament stress fibers with clusters of vinculin at the periphery (Figure 6A). Anti-β1 cells were rounded in shape with weaker actin staining and membrane-associated vinculin (Figure 6A). Loss of β1 integrin membrane distribution was determined in the anti-β1 group (Figure 6B), along with a reduction of Bag3 phosphorylation signals compared to controls (Figure 6C). Although there was no change of Munc18-1 protein level in anti-β1 cells (Figures 7A,B), anti-β1 treated cells showed reduced Snap25 and Vamp2 staining intensity and protein levels compared to controls (Figures 7C–F). This evidence indicates blocking β1-integrin suppresses collagen IV-INS-1 cell interactions and impairs subsequent downstream signaling and SNARE proteins that are required for insulin exocytosis. Furthermore, anti-β1 cells showed little to no colocalization of Snap25 and Vamp2 during glucose stimulation (Figure 8A), and diminished collagen IV induced high rate of insulin secretion at five or 60 min of high glucose stimulation (H5, H60) when compared to control groups (Figure 8B **).**

## Discussion

This study reveals the influence of COL IV and β1-integrin interactions on INS-1 cell exocytotic proteins and the subsequent augmentation in glucose-stimulated insulin secretion (GSIS). INS-1 cells cultured on COL IV were found to have significantly increased adhesion, spreading and focal adhesions. This was associated with enhanced immunofluorescent staining of β1-integrin, Bag3, and SNARE proteins. Underscoring the role of β1-integrin in these results, blockade of β1-integrin diminished focal adhesions, reduced SNARE protein staining and colocalization, and decreased insulin secretion. In reference to cell-based therapies for diabetics, this study suggests specific collagen IV-β1-integrin interactions are critical for proper beta-cell insulin secretion and should be further explored for utilization in cell-therapies techniques.

Consistent with results of previous studies, collagen IV was found to induce beta cell adhesion and spreading, and increased focal adhesions markers actin and vinculin (Kaido et al., 2004; Krishnamurthy et al., 2008). Focal adhesions are sites of contact between extracellular matrix proteins and the cytoskeleton mediated by integrins (Wozniak et al., 2004; Wu, 2007). In beta-cells, focal adhesions are documented to play many roles in development and mature function especially GSIS (Hammar et al., 2004; Saleem et al., 2009; Rondas et al., 2012; Townsend and Gannon, 2019). The present study supports previous work from our lab and other groups that demonstrated collagen IV increases focal adhesions and augments GSIS (Kaido et al., 2004; Krishnamurthy et al., 2008; Krishnamurthy et al., 2011). These effects were determined to be induced, at least in part, via β1-integrin. Blocking of β1-integrin reduced β1-integrin membrane-associated localization without impacting total protein levels (data not shown). Ultimately this lack of activation significantly decreased INS-1 cell adhesion and spreading, and diminished formation of focal adhesions and subsequent signaling. Compared to control, anti-β1 cells were rounded in shape, and had weaker actin staining and membrane-associated vinculin compared to controls. Anti-β1 cells also displayed significantly decreased basal and glucose-stimulated insulin secretion. Decreased basal insulin secretion has been observed in a beta-cell specific β1-integrin knockout mouse model (Peart et al., 2017), and anti-β1 treated INS-1 cells have been demonstrated to have decreased cell viability (Krishnamurthy et al., 2008). Therefore, decreased basal insulin secretion could be mediated via decreased cell viability and/or linked to the loss of β1-integrin signaling. Decreased glucose-stimulated insulin secretion is supported by previous *in vivo* (Riopel et al., 2011; Peart et al., 2017) and *in vitro* (Krishnamurthy et al., 2008; Krishnamurthy et al., 2011) work from our lab as well as other work (Diaferia et al., 2013). Furthermore, there was no change in the cell insulin content, indicating that the reduced of insulin secretion with β1-integrin blocking is linked to impaired insulin exocytotic, but not insulin synthesis.

There is a great deal of research supporting alterations in exocytotic proteins impacting exocytosis (Spurlin and Thurmond, 2006; Jeans et al., 2007; Oh et al., 2012), yet the relationship between cell-ECM linked to β1-integrin interactions on exocytotic proteins is only minorly explored (Fernández -Montes et al., 2011; Hellwig et al., 2011). The purpose of this study was to expand on previous research by exploring on the idea that collagen IV, through β1-integrin, also induces changes in insulin secretion via changes in exocytotic machinery. Bag3, a regulator of Snap25 and insulin secretion that is phosphorylated by FAK (Iorio et al., 2015), was demonstrated to be slightly increased, although not significantly, in total protein and phosphorylation in INS-1 cells cultured on collagen IV, thus impacting regulation of available Snap25. Collagen IV was also found to increase Munc18-1, Snap25 and Vamp2 cellular intensity. Furthermore, increased Snap25 and Vamp2 colocalization was visually observed in COL IV cells compared to control. However, under a β1-integrin blocking antibody treatment, COL IV-promoted Bag3 phosphorylation, Snap25 and Vamp2 cellular intensity and colocalization was diminished, while no alterations in Munc18-1 were observed. This observation corroborates our previous report on an inducible beta-cell specific β1-integrin knockout mouse model study which showed that knockout of beta-cell β1-integrin significantly reduced Snap25 and Vamp2 mRNA and cellular staining signals (Peart et al., 2017). This study demonstrates that COL IV, through β1-integrin, may influence SNARE proteins involved in insulin secretion indirectly *via* Bag3 and directly via their localization, and interactions. However, given that Munc18-1 was not influenced by the β1-integrin block, there are also other mediators for collagen IV-induced effects in INS-1 cells.

Finally, colocalization of SNARE proteins Snap25 and Vamp2 were examined at different time points during glucose stimulated insulin secretion. Interestingly, following 5 minutes of high glucose stimulation (H5), COL IV cells displayed prominent colocalization of Snap25 and Vamp2 distributed at the cell membrane and in the cytoplasm correlating with an increase in insulin secretion rate compared to control. However, colocalization was not preserved at 30 min (H30) and slightly returned at 60 min (H60) of high glucose stimulation, corelating to insulin secretion rates. β1-integrin was determined to be responsible for these changes, as anti-β1-integrin treatment resulted in a considerable decrease of Snap25 and Vamp2 colocalization for all glucose stimulated conditions coinciding with decreased rate of insulin secretion 25–50% lower than that of controls. Co-immunoprecipitation of Snap25 and Vamp2 in membrane fractions has previously been utilized to identify the docked pool of insulin vesicles (Daniel et al., 1999).Thus, increased membraned colocalization of these proteins is indicative of increased docked granules for insulin release. There is also a notable amount of overlap within the cytoplasm. This has been demonstrated before in neurons where SNAP25 was found in synaptic vesicles with other SNARE proteins (Walch-Solimena et al., 1995). There are many potential functions for this overlap including replenishment of the readily releasable pool, protein recycling, sequential exocytosis, and fusion between vesicles (Walch-Solimena et al., 1995; Hays et al., 2020). More research is required to determine the mechanism behind the increase in colocalization.

In summary, the present study provides evidence of a multifaceted role of COL IV signaling via β1-integrin, specifically in the promotion of SNARE proteins expression linked to insulin secretion in INS-1 cells. Our research supports previous work and underscores the significance of collagen IV-β1-integrin interactions on beta-cell adhesion, spreading, focal adhesions, and insulin secretion. A potential pathway linking collagen IV-β1-integrin signaling to FAK induced Bag3 phosphorylation and alterations in SNARE proteins during glucose-stimulated insulin secretion has been identified. This study also provided evidence of β1-integrin influencing the colocalization of Snap25 and Vamp2 in connection to increased insulin secretion rates. Ultimately, understanding the ideal external environment that promotes essential integrin-ECM interactions in pancreatic beta-cells will be valuable in the optimization of cell-based therapies for the treatment of diabetes.

## Data Availability

The original contributions presented in the study are included in the article/Supplementary Material, further inquiries can be directed to the corresponding author.

## References

[B1] Arous C. Wehrle-Haller B. (2017). Role and Impact of the Extracellular Matrix on Integrin-Mediated Pancreatic β-cell Functions. Biol. Cell 109, 223–237. 10.1111/boc.201600076 28266044

[B2] Bosco D. Meda P. Halban P. A. Rouiller D. G. (2000). Importance of Cell-Matrix Interactions in Rat Islet Beta-Cell Secretion *In Vitro*: Role of Alpha6beta1 Integrin. Diabetes 49, 233–243. 10.2337/diabetes.49.2.233 10868940

[B3] Cai E. P. Casimir M. Schroer S. A. Luk C. T. Shi S. Y. Choi D. (2012). *In Vivo* Role of Focal Adhesion Kinase in Regulating Pancreatic β-Cell Mass and Function through Insulin Signaling, Actin Dynamics, and Granule Trafficking. Diabetes 61, 1708–1718. 10.2337/db11-1344 22498697 PMC3379666

[B4] Daniel S. Noda M. Straub S. G. Sharp G. W. (1999). Identification of the Docked Granule Pool Responsible for the First Phase of Glucose-Stimulated Insulin Secretion. Diabetes 48, 1686–1690. 10.2337/DIABETES.48.9.1686 10480595

[B5] Diaferia G. R. Jimenez-Caliani A. J. Ranjitkar P. Yang W. Hardiman G. Rhodes C. J. (2013). β1 Integrin Is a Crucial Regulator of Pancreatic β-cell Expansion. Development 140, 3360–3372. 10.1242/dev.098533 23863477 PMC3737718

[B6] Fernández-Montes R. D. Blasi J. Busquets J. Montanya E. Nacher M. (2011). Fibronectin Enhances Soluble N-Ethylmaleimide-Sensitive Factor Attachment Protein Receptor Protein Expression in Cultured Human Islets. Pancreas 40, 1153–1155. 10.1097/MPA.0B013E318222BCAF 21926557

[B7] Gupton S. L. Gertler F. B. (2010). Integrin Signaling Switches the Cytoskeletal and Exocytic Machinery that Drives Neuritogenesis. Developmental Cell 18, 725–736. 10.1016/j.devcel.2010.02.017 20493807 PMC3383070

[B8] Hammar E. Parnaud G. Bosco D. Perriraz N. Maedler K. Donath M. (2004). Extracellular Matrix Protects Pancreatic β-Cells against Apoptosis. Diabetes 53, 2034–2041. 10.2337/diabetes.53.8.2034 15277383

[B9] Hays C. L. Grassmeyer J. J. Wen X. Janz R. Heidelberger R. Thoreson W. B. (2020). Simultaneous Release of Multiple Vesicles from Rods Involves Synaptic Ribbons and Syntaxin 3B. Biophysical J. 118, 967–979. 10.1016/J.BPJ.2019.10.006 PMC7036726 31653448

[B10] Hellwig S. Hack I. Kowalski J. Brunne B. Jarowyj J. Unger A. (2011). Role for Reelin in Neurotransmitter Release. J. Neurosci. 31, 2352–2360. 10.1523/JNEUROSCI.3984-10.2011 21325502 PMC6623706

[B11] Howe G. A. Addison C. L. (2012). β1 Integrin. Cell Adhes. Migration 6, 71–77. 10.4161/cam.20077 PMC3499315 22568952

[B12] Hynes R. O. (2002). Integrins. Cell 110, 673–687. 10.1016/S0092-8674(02)00971-6 12297042

[B13] Iorio V. Festa M. Rosati A. Hahne M. Tiberti C. Capunzo M. (2015). BAG3 Regulates Formation of the SNARE Complex and Insulin Secretion. Cell Death Dis 6, e1684. 10.1038/cddis.2015.53 25766323 PMC4385931

[B14] Jeans A. F. Oliver P. L. Johnson R. Capogna M. Vikman J. Molnár Z. (2007). A Dominant Mutation in Snap25 Causes Impaired Vesicle Trafficking, Sensorimotor Gating, and Ataxia in the Blind-Drunk Mouse. Proc. Natl. Acad. Sci. U.S.A. 104, 2431–2436. 10.1073/pnas.0610222104 17283335 PMC1793901

[B15] Kaido T. Yebra M. Cirulli V. Montgomery A. M. (2004). Regulation of Human β-Cell Adhesion, Motility, and Insulin Secretion by Collagen IV and its Receptor α1β1. J. Biol. Chem. 279, 53762–53769. 10.1074/JBC.M411202200 15485856

[B16] Krishnamurthy M. Li J. Al-Masri M. Wang R. (2008). Expression and Function of αβ1 Integrins in Pancretic Beta (INS-1) Cells. J. Cell Commun. Signal. 2, 67–79. 10.1007/s12079-008-0030-6 19023675 PMC2648043

[B17] Krishnamurthy M. Li J. Fellows G. F. Rosenberg L. Goodyer C. G. Wang R. (2011). Integrin α3, but Not β1, Regulates Islet Cell Survival and Function via PI3K/Akt Signaling Pathways. Endocrinology 152, 424–435. 10.1210/en.2010-0877 21177833

[B18] Oh E. Kalwat M. A. Kim M.-J. Verhage M. Thurmond D. C. (2012). Munc18-1 Regulates First-phase Insulin Release by Promoting Granule Docking to Multiple Syntaxin Isoforms. J. Biol. Chem. 287, 25821–25833. 10.1074/jbc.M112.361501 22685295 PMC3406668

[B19] Peart J. Li J. Lee H. Riopel M. Feng Z.-C. Wang R. (2017). Critical Role of β1 Integrin in Postnatal Beta-Cell Function and Expansion. Oncotarget 8, 62939–62952. 10.18632/oncotarget.17969 28968961 PMC5609893

[B20] Plow E. F. Haas T. A. Zhang L. Loftus J. Smith J. W. (2000). Ligand Binding to Integrins. J. Biol. Chem. 275, 21785–21788. 10.1074/JBC.R000003200 10801897

[B21] Riopel M. Krishnamurthy M. Li J. Liu S. Leask A. Wang R. (2011). Conditional β1-integrin-deficient Mice Display Impaired Pancreatic β Cell Function. J. Pathol. 224, 45–55. 10.1002/path.2849 21381031

[B22] Rondas D. Tomas A. Soto-Ribeiro M. Wehrle-Haller B. Halban P. A. (2012). Novel Mechanistic Link between Focal Adhesion Remodeling and Glucose-Stimulated Insulin Secretion. J. Biol. Chem. 287, 2423–2436. 10.1074/jbc.M111.279885 22139838 PMC3268403

[B23] Saleem S. Li J. Yee S.-P. Fellows G. F. Goodyer C. G. Wang R. (2009). β1 integrin/FAK/ERK Signalling Pathway Is Essential for Human Fetal Islet Cell Differentiation and Survival. J. Pathol. 219, 182–192. 10.1002/path.2577 19544355

[B24] Spurlin B. A. Thurmond D. C. (2006). Syntaxin 4 Facilitates Biphasic Glucose-Stimulated Insulin Secretion from Pancreatic β-Cells. Mol. Endocrinol. 20, 183–193. 10.1210/me.2005-0157 16099818

[B25] Stupack D. G. Cheresh D. A. (2002). Get a Ligand, Get a Life: Integrins, Signaling and Cell Survival. J. Cell Sci 115, 3729–3738. 10.1242/jcs.00071 12235283

[B26] Townsend S. E. Gannon M. (2019). Extracellular Matrix-Associated Factors Play Critical Roles in Regulating Pancreatic β-Cell Proliferation and Survival. Endocrinology 160, 1885–1894. 10.1210/en.2019-00206 31271410 PMC6656423

[B27] Walch-Solimena C. Blasi J. Edelmann L. Chapman E. R. von Mollard G. F. Jahn R. (1995). The T-SNAREs Syntaxin 1 and SNAP-25 Are Present on Organelles that Participate in Synaptic Vesicle Recycling. J. Cell Biol 128, 637–645. 10.1083/jcb.128.4.637 7860636 PMC2199899

[B28] Wang R. Li J. Lyte K. Yashpal N. K. Fellows F. Goodyer C. G. (2005). Role for β1 Integrin and its Associated α3, α5, and α6 Subunits in Development of the Human Fetal Pancreas. Diabetes 54, 2080–2089. 10.2337/diabetes.54.7.2080 15983209

[B29] Wang R. Wang R. (2009). Integrins and Extracellular Matrices in Pancreatic Tissue Engineering. Front. Biosci. S1, 477–491. 10.2741/s39 19482715

[B30] Wozniak M. A. Modzelewska K. Kwong L. Keely P. J. (2004). Focal Adhesion Regulation of Cell Behavior. Biochim. Biophys. Acta (Bba) - Mol. Cell Res. 1692, 103–119. 10.1016/J.BBAMCR.2004.04.007 15246682

[B31] Wu C. (2007). Focal Adhesion: a Focal point in Current Cell Biology and Molecular Medicine. celladhesion 1, 13–18. 10.4161/cam.408110.4161/cam.1.1.4081 PMC2633675 19262093

[B32] Yashpal N. K. Li J. Wheeler M. B. Wang R. (2005). Expression of β1 Integrin Receptors during Rat Pancreas Development-Sites and Dynamics. Endocrinology 146, 1798–1807. 10.1210/en.2004-1292 15618357

