# Supplementary material for: Collagen IV-β1-Integrin Influences INS-1 Cell Insulin Secretion via Enhanced SNARE Protein Expression
Source: Front Cell Dev Biol. 2022 Apr 28;10:894422. doi: 10.3389/fcell.2022.894422 (PMC9096118; doi:10.3389/fcell.2022.894422)
Supplement: Supplementary file 1 [file Table1.DOCX]

Supplementary Table S1. Antibodies for immunohistochemistry/immunofluorescence and western blot analyses

| Primary Antibody | Dilution | Company |
| --- | --- | --- |
| Mouse Anti-BAG3 | 1:50/1:1000^W^ | Santa Cruz Biotechnology Inc, Dallas, TX, USA |
| Rabbit Anti-pBAG3 (Tyr457) | 1:^­^50 | Antibodies-online Inc., Limerick, PA, USA |
| Rabbit Anti-β1-integrin | 1:1000^W^ | Millipore Sigma, Saint Louis, MO, USA |
| Mouse Anti-β1-integrin | 1:50 | Abcam Inc., Cambridge, MA, USA |
| Rabbit Anti-FAK | 1:2000^W^ | Invitrogen, Burlington, ON, CA |
| Rabbit Anti-pFAK (Tyr397) | 1:1000^W^ | Invitrogen, Burlington, ON, CA |
| Mouse Anti-GAPDH | 1:2000^W^ | Santa Cruz Biotechnology Inc, Dallas, TX, USA |
| Rabbit Anti-Munc18 | 1:100*/1:1000^W^ | Abcam Inc., Cambridge, MA, USA |
| Phalloidin | 1:25 000 | Abcam Inc., Cambridge, MA, USA |
| Mouse Anti-SNAP25 | 1:50/1:1000^W^ | Santa Cruz Biotechnology Inc, Dallas, TX, USA |
| Mouse Anti-Syntaxin 1a | 1:50* | Santa Cruz Biotechnology Inc, Dallas, TX, USA |
| Rabbit Anti-VAMP2 | 1:200/1:500^W^ | Abcam Inc., Cambridge, MA, USA |
| Anti-Vinculin | 1:50 | Millipore Sigma, Saint Louis, MO, USA |
|  |  |  |
| Secondary Antibody | **Dilution** | **Company** |
| Goat Anti-Mouse | 1:50 | Jackson Immunoresearch, West Grove, Pam USA |
| Goat Anti-Rabbit | 1:50 | Jackson Immunoresearch, West Grove, Pam USA |
| Anti-Mouse HRP-linked | 1:500-3000^W^ | Cell Signaling Technology, Whitby, ON, CA |
| Anti-Rabbit HRP-linked | 1:500-3000^W^ | Cell Signaling Technology, Whitby, ON, CA |

* Citrate Antigen Retrieval (pH 6.0) used; ^W^Used in western blot probing.
